# Sleep Quality in Family Caregivers of Persons Living With Dementia

**DOI:** 10.1097/jnr.0000000000000587

**Published:** 2023-11-28

**Authors:** Yeu-Hui CHUANG

There are more than 55 million persons living with dementia (PwD) in the world, with around 10 million new cases identified each year (World Health Organization, 2023). This population relies heavily on family or other unpaid caregivers, who are often their spouse/partner or adult children, and requires high levels of care to continue living in the community due to their gradual loss of independent functioning. The informal care hours required by PwD living at home amount to around 2,089 hours per year (around 6 hours per day) per PwD (Wimo et al., 2018). The demands of caregiving are increasingly stressful, heavy, and exhausting as the disease progresses over time, and, consequently, impact the physical and psychological health of family caregivers (Alzheimer’s Association, 2023; Engel et al., 2022; Ma et al., 2018).

Sleep problems, including sleep disturbance, insomnia, and poor sleep quality, are common health-related issues frequently observed in family caregivers of individuals living with dementia. The results of a systematic review and meta-analysis study revealed that caregivers had 2.42 to 3.5 hours less sleep per week and poorer sleep quality than age-matched, non-caregiver controls (Gao et al., 2019). The sleep-related challenges faced by family caregivers may lead to adverse physical and psychological outcomes, poor quality of life, and compromised care for care recipients. Therefore, understanding the sleep quality experienced by caregivers and associated factors is imperative for assisting healthcare professionals to develop early and tailored interventions to improve sleep quality in family caregivers of PwD.

In this issue of *The Journal of Nursing Research*, Chu and Jang present evidence of poor sleep quality in spouse caregivers of PwD and also show that depressive symptoms significantly influence sleep quality in caregivers. Their findings emphasize the need for healthcare professionals to regularly check caregivers’ depressive symptoms and quality of sleep.

Beyond the article on sleep quality in spouse caregivers, this issue also covers a variety of topics from care for individuals in the community, pregnant women, and patients to nurse salaries, workplace violence, and the values of nursing professionals. Yang et al. used a qualitative approach to explore the experiences of family caregivers who provide care to older patients with cancer at home. Chang et al., using a randomized controlled trial research design, investigated the feasibility of three different exercise modalities, including aerobic exercise, aerobic exercise combined with resistance exercise, and high-intensity interval training, on improving body composition, anthropometric parameters, and lipid profiles in community-dwelling residents with physical inactivity. A systematic review and meta-analysis conducted by Sari, Hsu, and Nguyen assessed the effectiveness of a mindfulness-based intervention on mental health outcomes in pregnant women. Yilmaz Eker and Yilmaz’s randomized controlled study evaluated the effectiveness of the normothermia checklist in improving inadvertent perioperative hypothermia in patients undergoing surgery. In addition, Chang et al. compared nurse salaries between Organization for Economic Co-operation and Development (OECD) countries and Taiwan, and examined the factors associated with nurse salaries in OECD countries. Hsieh et al. conducted a qualitative study to understand the perceptions and coping strategies of nurse victims of workplace violence who were still working in emergency departments. Abu-El-Noor, Abu-El-Noor, and Allari assessed and compared professional nursing values among Jordanian and Palestinian undergraduate nursing students.

We are confident that the diverse range of papers in this issue not only offers readers up-to-date knowledge applicable to their practice but will also inspire further investigation and innovation.

## References

[bib1] Alzheimer’s Association. (2023). *2023 Alzheimer's disease facts and figures*. https://www.alz.org/media/Documents/alzheimers-facts-and-figures.pdf 10.1002/alz.13016 36918389

[bib2] Engel L. Loxton A. Bucholc J. Muldowney A. Mihalopoulos C., & McCaffrey N. (2022). Providing informal care to a person living with dementia: The experiences of informal carers in Australia. *Archives of Gerontology and Geriatrics*, 102, Article 104742. 10.1016/j.archger.2022.104742 35671552

[bib3] Gao C. Chapagain N. Y., & Scullin M. K. (2019). Sleep duration and sleep quality in caregivers of patients with dementia: A systematic review and meta-analysis. *JAMA Network Open*, 2(8), Article e199891. 10.1001/jamanetworkopen.2019.9891 31441938 PMC6714015

[bib4] Ma M. Dorstyn D. Ward L., & Prentice S. (2018). Alzheimers' disease and caregiving: A meta-analytic review comparing the mental health of primary carers to controls. *Aging & Mental Health*, 22(11), 1395–1405. 10.1080/13607863.2017.1370689 28871796

[bib5] Wimo A. Gauthier S. Prince M., & on behalf of ADI’s Medical Scientific Advisory Panel, and the Alzheimer’s Disease International publications team. (2018). *Global estimates of informal care*. https://www.alzint.org/u/global-estimates-of-informal-care.pdf

[bib6] World Health Organization. (2023). *Dementia*. Retrieved October 5, 2023, from https://www.who.int/health-topics/dementia#tab=tab_2

